# Coincidence analysis: Identifying factors contributing to frontline health workers’ confidence to face future pandemics across Honduras, Syria, and South Sudan

**DOI:** 10.1371/journal.pgph.0006925

**Published:** 2026-07-31

**Authors:** Maryada E. Vallet, Christopher G. Kemp, Husam Al Zuwayny, Erin M. Sorrell, Gilbert Burnham

**Affiliations:** 1 TANGO International, Tucson, Arizona, United States of America; 2 Department of International Health, Johns Hopkins Bloomberg School of Public Health, Baltimore, Maryland, United States of America; 3 Department of Environmental Health, Center for Health Security, Johns Hopkins Bloomberg School of Public Health, Baltimore, Maryland, United States of America; PLOS: Public Library of Science, UNITED STATES OF AMERICA

## Abstract

Frontline health workers in humanitarian settings play a critical role in pandemic response, yet evidence on what sustains response readiness beyond the emergency is limited. This study examined factors associated with primary healthcare workers’ sustained self-reported knowledge, skills, and confidence to respond to future outbreaks or pandemics, with the configurational analysis focused on confidence. A retrospective cross-sectional survey was conducted among 118 health workers in Honduras, Syria, and South Sudan who received COVID-19 training. Coincidence analysis was applied with a set of associated predictors to identify combinations of conditions linked to high sustained confidence. COVID-19 training that covered five or more core topics was associated with high sustained self-rated capacities; topics included proper use of personal protective equipment, transmission routes, procedures for case screening, and facility infection prevention and control. Continuity of primary healthcare services after emergency funding ended, fewer disruptions from conflict or natural disasters, and supportive team structures were linked to confidence. Coincidence analysis indicated multiple sufficient causal pathways to sustained confidence, the first being very high self-rated post-training knowledge as a standalone pathway (consistency: 0.66, coverage: 0.62). Other pathways included health workers with higher education combined with either case-screening training or working in facilities that continued services post-funding (consistency: 0.604, coverage: 0.64). Applying COVID-19 practices to other outbreaks (e.g., dengue, cholera) further reinforced capacities and illustrated the pathogen-agnostic value of core infection prevention and response competencies. Results underscore that durable preparedness at the primary healthcare level in fragile contexts depends on comprehensive, practice-oriented training for all types of frontline health workers, as well as enabling conditions that maintain service delivery. This study contributes to emerging literature using implementation science frameworks and coincidence analysis to understand complex causal pathways. The findings should inform global health security investments, including in the world’s most fragile health systems.

## Introduction

Ensuring that countries with humanitarian emergencies and fragile health systems are adequately prepared to respond to outbreaks at the community and primary healthcare (PHC) level remains a critical global health gap [1–3], especially as future pandemics are on the horizon [4,5]. The Coronavirus Disease 2019 (COVID-19) pandemic highlighted both the critical role of frontline health workers and the need to address vulnerabilities and build resilience of PHC systems [6–9]. Frontline health workers in humanitarian settings face unique challenges in building pandemic capacities within fragile and fragmented healthcare infrastructures, which are degraded from recurrent natural disasters and insecurity combined with chronic under-resourcing [3,10–12]. The COVID-19 pandemic provided a surge of resources for just-in-time health worker trainings and support, as well as an opportunity for learning around health emergency capacity building. While a companion study using the same dataset documented that pandemic capacities were retained two to three years post-training [13], the interplay of factors driving that sustainment remains unexamined. By identifying the factors that contribute to sustained confidence of health workers to face future outbreaks or pandemics across three geographic regions, this research aims to inform approaches for better incorporating humanitarian contexts in existing Global Health Security (GHS) frameworks and strategies, including the International Health Regulations (IHR).

There is literature examining health worker knowledge, attitudes, and practices related to COVID-19 training in resource-limited and humanitarian settings including Ethiopia, Nigeria, and Uganda [14–19]. Yet, this research has focused on immediate post-training outcomes rather than longer-term sustainment. Studies highlight various factors influencing knowledge, attitudes, and practice as post-training outcomes across individual, intervention (training), health facility, and contextual dimensions. Individual factors such as health worker gender have been associated with differing levels of pandemic readiness, with some studies reporting improved infection prevention and control (IPC) practices among female health workers [15], others highlight advantages among male health workers [14,17,18]. Improved knowledge, attitude, and practice are associated with higher levels of education, or specifically, physician roles [14,16–19]. Access to COVID-19 training and IPC guidelines have also shown relationships with improved self-efficacy and infection prevention practices [15,17]. Further, the practice setting and contextual factors—such as prior experience with disease outbreaks (e.g., Ebola), facility type, job satisfaction, and supervisory support—are reported as contributors to humanitarian health worker confidence and perceived readiness during COVID-19 [18,19].

There remains a critical gap in understanding which factors interact, and how, to sustain humanitarian health worker capacities over time to face future outbreaks and pandemics. This study examines factors associated with health workers’ perceived knowledge, skills, and confidence to face infectious disease risks two to three years post-training. The analyses and discussion for this paper focus on causal pathways for health worker confidence. Medical education and training commonly use self-assessment tools with constructs including knowledge, skills or practice performance, and confidence or self-efficacy [20,21]. Confidence in relation to medical professional education refers to an attitude or belief of one’s ability to effectively apply learned knowledge or skills [22,23]. The confidence outcome was the focus of this study’s model as the literature suggests the capacities may be nested—in that knowledge supports better practice, which supports perceptions of capability or confidence to handle emerging disease threats [15,16,24–26].

This study helps to address the literature gap by providing learning across regions, conducted in three distinct humanitarian contexts: Honduras, Syria (both regime-controlled and northern regions), and South Sudan, where increased international assistance was provided during the pandemic [27]. Descriptions of the country selection criteria and country case study contexts are provided in the “sample and setting” section below. The study utilized data from a COVID-19 Evaluation (2020–2022) for the formerly known United States Agency for International Development/Bureau for Humanitarian Assistance (USAID/BHA).

## Materials and methods

### Design and instrument

This study design was retrospective cross-sectional utilizing a semi-structured survey with health workers in Honduras, Syria, and South Sudan. The study objective was to identify the factors contributing to health worker perceptions of sustained pandemic preparedness. It examined individual, COVID-19 training characteristics, practice setting, and context factors. The target population included community and PHC-level health workers who received COVID-19 training and support from humanitarian partners between 2020 and 2022 with funding from USAID/BHA. The full descriptive results from this dataset are reported in a companion paper [13].

There were no standardized measures or existing validated tools that fit the study objective, humanitarian context, and the new COVID-19 realities, which limits the comparability to other research [28]. For instance, validated knowledge, attitude, and practice tools adapted for COVID-19 were too long to fit within study timing constraints [18] or focused only on specific IPC knowledge [29]. For this study, a survey module tailored to the research question and program context was included in the larger tool for the overall USAID/BHA COVID-19 Evaluation. The module included structured and unstructured questions.

#### Conceptual framework.

The survey indicators were developed using examples from literature and mapped within the implementation research domains of the Dynamic Sustainability Framework (DSF) [30]. The DSF captures changes in the intervention benefits over time while considering individual and training characteristics and the context at the practice and system levels. The study outcomes comprised self-reported capacity measures of knowledge, skills, and confidence pre-training (T0, Time Zero), during/post-training (T1, Time One), and at the time of data collection (T2, Time Two) in March/April 2024. This provides a retrospective assessment of the training’s perceived sustained effects, as the data collection occurred nearly three years (median of 34 months) after the COVID-19 trainings. Outcome indicators were used on a five-point rating scale [31].

**Fig 1:**
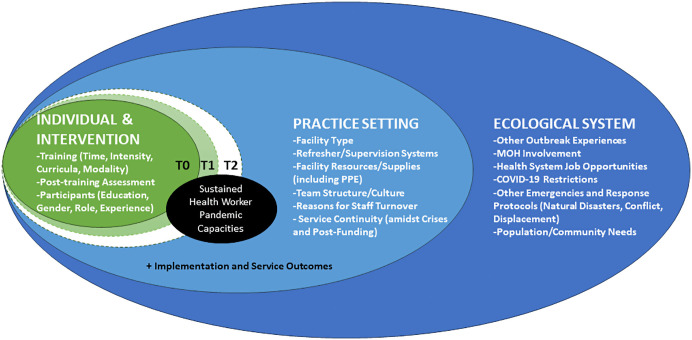
Adapted dynamic sustainability framework for sustained health worker capacities.

Fig 1 shows the adapted domains of this framework [30]. Intervention components assessed included training duration, delivery modality, coverage of topics, and post-training assessment. Training participant characteristics included gender, role, education, and years of experience. The practice setting included type of facility (i.e., mobile medical unit), supervision or refresher training opportunities, access to critical supplies, and staff/team structures. The ecological system domain included involvement or support from the Ministry of Health (MoH), service and job continuity post-funding, other outbreak experience, and other concurrent emergencies. Constructs for implementation and service outcomes were also included in the framework as they relate to sustaining the outcomes and institutionalization, including: adoption (utilizing proxy measure of post-training knowledge), penetration at health worker level (utilizing proxy of reported access to refresher training), sustainability (facility-level institutionalization using health worker reported support from facility), equity (preparedness gaps based on gender), and satisfaction with the training [32].

#### Ethics statement.

The survey instrument was reviewed by international and country-based health and evaluation technical experts. It was translated into Spanish (Honduras) and Arabic (Syria), while the original English version was retained for South Sudan, where research team training ensured practice for simultaneous translation to local dialects as needed. Translated tools were pilot-tested by local health professionals and programmed for tablet-based data collection.

Study procedures including the consent process were reviewed by the Johns Hopkins Bloomberg School of Public Health Institutional Review Board (IRB), which determined that the study did not constitute human subjects research. Verbal informed consent was obtained from all survey participants prior to data collection. Enumerators read a standardized consent script, asked participants to restate the study purpose and procedures to confirm understanding, and documented the consent process. In each participating country/location, USAID representatives confirmed that national IRB review was not required and provided country-level oversight. Additional permissions and coordination were secured from relevant local authorities for each geography and government ministries by the national research partners: ANED Consultores (Honduras), Trust Consultancy and Development (Syria), and Specialized Logistics Solutions (South Sudan).

**Inclusivity in global research**: Additional ethical, cultural, and scientific considerations specific to inclusivity in global research may be found in the Supporting Information (S1 Checklist).

### Sample and setting

#### Sample size calculation.

The required sample size was determined using the Cochran formula for a single proportion, suited to descriptive cross-sectional studies with ordinal outcome measures (knowledge, skills, and confidence assessed on a five-point scale) [33,34]. With maximum variability assumed (*p* = 0.50), a 95% confidence level (*Z* = 1.96), and a margin of error of 10% (*e* = 0.10), a minimum of 96 participants was established.

#### Country selection and context.

A multi-stage non-probability sampling strategy was used, beginning with purposive case country and partner health facility site selection. The study focused on Honduras, Syria, and South Sudan, representing three USAID/BHA-funded regions selected for their high level of funding within the region, accessibility, complex emergency typology, relevant health sector funding, and partner availability. A target of 30–35 completed surveys per country was established across selected sites, reflecting the recognized minimum threshold for meaningful subgroup disaggregation [35]. In Syria, the distinct governance and operational structures across regime-controlled and non-regime-controlled areas necessitated treating each as a separate sampling region. This methodology was designed to yield a contextualized snapshot of workforce conditions and dynamics within each setting. Within each country, the health partners purposively selected health facilities based on logistical and security feasibility.

See Table 1 for a context summary of each country. South Sudan represents a fragile, post-conflict state severely lacking health infrastructure, where the vast majority of health services are delivered by international organizations to remote and displaced populations facing compounding crises of recurrent flooding, severe food insecurity, and multiple disease outbreaks. Syria represents a protracted civil war setting that has decimated health infrastructure and workforce capacity, and the COVID-19 response was further complicated by fragmented territorial governance, a major earthquake, and concurrent epidemic-prone disease outbreaks. As shown in the table below, Syria has an exceptionally low rate of COVID-19 vaccination, attributed to inequity in distribution and vaccine hesitancy [36,37]. Honduras represents a lower-middle-income country with a chronically underfunded public health system that was simultaneously managing COVID-19, back-to-back Category 4 hurricanes displacing hundreds of thousands from health services, insecurity from gangs, and endemic vector-borne disease threats.

**Table 1:** Humanitarian context by case study country.

Country Context 2020-2023	South Sudan [38–41]	Syria [42–46]	Honduras [47–49]
**Emergency types (conflict + natural disaster)**	Protracted factional conflict and consecutive floods, causing mass internal displacement and severe food insecurity	Protracted conflict/civil war and fractured control across regions, mass displacement (internal & external), February 2023 earthquake in northwest	Ongoing insecurity from gang violence, and two hurricanes (November 2020)
**Other concurrent outbreaks**	Numerous outbreaks (Typhoid, Hepatitis E Virus, Malaria, Measles, Cholera)	Numerous outbreaks (Cholera, Meningitis, Measles, Hepatitis A)	Dengue (2019 national emergency/ongoing)
**Confirmed COVID-19 deaths (per 100,000) per available data** [50]	1.334	14.081	106.213
**Percent of population with at least one dose of COVID-19 vaccine by August 2024**	39.2%	14.7%	63.1%
**Health system context for survey**	Humanitarian Partners: Two INGOs; Extremely limited health system	Mixed: Three UN agencies’ health centers with local partners (Regime-controlled centers not accessible)	Public: UN agency support to public system community health centers
**GHS Index (of 195 countries)** [51]	185th	192nd	163rd

Table acronyms: INGOs, International Non-Government Organizations; UN, United Nations; GHS, Global Health Security.

#### Participant selection.

Eligible participants included all health project workers engaged in community or primary healthcare services who had received COVID-19 training between 2020 and 2022. Exclusion criterion, therefore, were health workers from secondary or tertiary care facilities, who were not employed in primary healthcare services during those pandemic years, or who did not receive COVID-19 training. To reduce selection bias, workers were randomly selected from those available and consenting at the time of data collection, and characteristics were compared to trained but unavailable workers. Partners confirmed no systematic differences between sampled and non-sampled health workers.

At each site, the number of trained workers was identified, followed by those still employed and verbally consenting to participate. Where more workers were available than required for the sample, and to reduce selection bias, random sampling was used. The sample recruitment dates for each country were: 12/03/2024 – 18/04/2024 for Honduras; 04/04/2024 – 30/04/2024 for Syria; 16/03/2024 – 28/03/2024 for South Sudan. No selected survey participants declined participation. To assess for selection bias around the available/employed versus unavailable health workers, trained health workers who were not available for the survey were compared against respondents across key individual characteristics—including healthcare role, gender, and ethnicity. This confirmed that the sample was not systematically different from the broader trained population; this methodological consideration is discussed further in the limitations section.

#### Data collection and management.

Prior to data collection, a standardized training protocol was implemented across all countries to ensure consistency in instrument administration. Country research leads each conducted a four-hour virtual training-of-trainer session with the local research partner, followed by their facilitation of a one-day in-person workshop for their respective local research teams. Training content, approved by the principal investigator (PI), covered instrument walkthrough, operational definitions of key terms across contexts, and informed consent and interview procedures. To mitigate recall bias and social desirability, data collectors administered verbal privacy assurances at the start of each interview, explicitly informing participants that their individual responses would remain confidential and would not be shared with facility supervisors or project partners. To support accurate recall, participants were provided contextual reminders at the beginning of the survey: interviewers briefly described the COVID-19 training period and implementing partners, referenced key events or outbreaks in their country context, and asked participants to orient to that time before responding. Survey translation and instrument refinements are noted in the section above on expert and ethical review. A single instrument version was administered across all countries to ensure cross-country comparability.

The PI served as the central supervisory lead throughout the data collection period. Operational oversight was conducted across multiple levels: research partners provided daily on-site supervision, local supervisors held weekly check-ins with country research leads, and ongoing spot-checks were conducted as data were uploaded to KoBo Toolbox to identify and resolve issues in real time. Following completion of fieldwork, structured debriefs were conducted with each country team. Full country datasets were reviewed jointly by the country research lead and PI immediately upon submission to address any outstanding queries prior to analysis. Data cleaning was conducted across the complete dataset by the team analyst according to a plan developed and approved by the PI. Research teams in each region ensured safety, anonymity, and confidentiality of participants during the data collection, and data were uploaded to secure servers.

### Analysis procedures

Configurational Comparative Methods (CCM), specifically Coincidence Analysis (CNA), offer significant advantages for analyzing complex real-world implementation research in humanitarian settings. CCM systematically compares cases to identify combinations of conditions, such as implementation strategies and contextual factors, that influence outcomes [52]. CNA develops binary variables and tests models to identify necessary and sufficient conditions for outcomes [53–55]. Key benefits include its suitability for small to medium sample sizes, ability to integrate qualitative and semi-structured responses, and capacity to uncover causal complexities without relying on controlled trial designs [55,56]. CNA identifies combinations of explanatory factors, equifinality (multiple successful intervention pathways), and conjunctural causation (interactive effects of multiple conditions) [55,56]. Additionally, CNA is adept at managing data imbalances and some levels of data noise, ensuring robust causal inference even in challenging data collection environments [57].

The first preparatory step involved defining, calibrating, and selecting the outcome and condition variables. Data preparation was done using Stata/SE 18 [58]. This process started with identifying relevant cases from the health worker survey dataset. Next, thresholds or breakpoints were established through sensitivity analyses, examining variable distributions (e.g., via histograms and detailed summaries) to identify natural breaks [55]. Tabulation with the outcomes followed to ensure empirical diversity and to avoid sparse cell representation, with recommended minimum thresholds of 10–20% for any given configuration [55,59]. All variables were then tested for association with the outcomes using Chi-square tests.

To better understand potential hierarchical effects of the dataset overall, intra-class correlation tests were conducted, revealing clustering effects at the health facility level rather than country level for the skills and confidence outcomes. A benefit of CNA is its ability to handle multi-level causal interactions and clustering [54], so no facility-level variable was included in the model. The three outcome variables were highly correlated (*p* < 0.000). Thus, the CNA model focused on the confidence outcome.

Following initial data preprocessing, Chi-square (*p* < 0.10), and empirical diversity checks, the first set of variables to be considered for inclusion were tested for redundancy and association as a Confidence model using pairwise correlation analyses and logistic regression models (*p* < 0.05). This was used to ensure potential suitability for causal inference and to further reduce the number of predictors [54,55,60]. A maximum of six Confidence model predictor variables were identified as: statistically significantly associated with the outcome (*p* < 0.05), with strong theoretical justification, without high correlation between variables, and/or that remained in the stepwise logistic regression model.

The CNA analysis was conducted using the R package (version 3.6.2) [61,62]. The dataset was pre-processed using a configuration table to examine the diversity of configurations and ensure compatibility with CNA’s logical requirements. The analysis adhered to best practices for CCM, balancing consistency and coverage thresholds [54–56]. Initial models were run with default consistency and coverage thresholds (0.8) but no solutions were found. Thresholds for consistency and coverage were systematically varied (e.g., 0.7, 0.6) to account for sparse configurations and maximize the identification of causal pathways that recurred at varying thresholds as a robustness check [54]. Maxstep parameters were to set (3,3,9) to help minimize under or overfitting of complex models [60]. Equifinality—the presence of multiple causal pathways leading to the same outcome—was evident in the analysis, with different combinations of conditions explaining high confidence ratings [55]. An asymmetrical model was tested to assess for the absence of confidence, but those results are not presented here.

The survey included open-ended questions that were coded as structured thematic or binary responses. This coding was completed separately by two team members. Inter-coder consistency was checked and discrepancies agreed with the PI. A selection of quotes from open-ended responses complementing the quantitative survey findings are provided in the results.

## Results

### Sample overview

The final survey sample (n = 118) includes all health workers who received a COVID-19 training and reported all self-rated outcomes; this sample is shown in Table 2 by percent of the sample who were medical doctors and male/female, and by country/ location, number and type of health partners and health centers.

**Table 2:** Health worker CNA sample overview.

Country/Location	Number and Type of Partners	Number of health centers	Percent Medical Doctors	Male/Female	n=
Honduras (Atlántida, Cortés, Yoro Regions)	1 UN/ Government of Honduras	14	47.8	2/21	23
Syria: GOS (Hama, Damascus Governorates)	3 UN	8	19.4	9/27	36
Syria: NS (Hasaka, Idleb, northern Aleppo)	3 UN	6	5.1	24/15	39
South Sudan (Upper Nile and Warrap States)	2 NGO	11	0.0	14/6	20
	**9 Projects**	**39 Facilities**	**16.9%**	**49/69**	**118**

The Honduras survey included health workers across eight departments of three regions, the majority in the hurricane-affected region of Cortes. The United Nations (UN) project supported 32 Government of Honduras PHC centers, with this analysis sample comprising 23 health workers (91.3% female, 47.8% doctors) across 14 health centers. The Syria sample in Government of Syria (GOS) included 36 health workers (75% female, 19.4% doctors) across eight facilities, and 39 health workers from Northeast/northwest Syria (NS) (61.5% male, 5.1% doctors) across six facilities. The Syria survey covered projects by three UN agencies implemented through their local non-governmental organization (NGO) partners, with facilities in both regions affected by the earthquake and ongoing conflict. South Sudan interviews were conducted in the remote Upper Nile (e.g., Kodok, Melut, Malakal) affected by recurrent floods, covering 11 PHC centers run by two international NGOs (INGOs). Due to the isolated nature of the area, the South Sudan sample (n = 20) was majority male (70.0%) and all non-physicians.

### Predictor associations with outcomes

The results of the chi-square analyses revealed several statistically significant associations between predictor variables and the knowledge, skills, and confidence outcomes among health workers. The binary outcomes represent “very high” time-of-survey self-reported knowledge (of the IPC-related COVID-19 training topics), skills (to handle new disease risks), and confidence (to apply knowledge and skills to new disease risks). Table 3 shows those predictors organized by conceptual model domain with *p* < 0.10 associations with any of the outcomes and/or substantial theoretical justification.

**Table 3:** Predictor associations with current “very high” knowledge, skills, confidence.

Predictor Variable ^a^	Knowledge p-value	Skills p-value	Confidence p-value	Theoretical justification for model: supporting literature or relevance to conceptual model	Consider for confidence model? ^b^
**Individual Characteristics**					
Experience binary - six or more years Experience categorical Low (0–5 years) Medium (6–10 years) High (11 or more years)	0.042** 0.108	0.059* 0.150	0.909 0.977	Supporting literature for knowledge/skills [15–19,63,64] Conceptual model: Participant years of experience in healthcare	Exclude
Education binary - University or higher Education categorical: Secondary or less; Technical school or some college; University degree completed; Graduate training	1.000 0.408	0.877 0.750	0.965 0.934	Substantial supporting literature [10,15,16,63–65] Conceptual model: Participant level of education	Include
Clinical roles categorical Doctor/Clinic Director; Nurse; Other Clinical; Administrative; Community worker/outreach	0.062*	0.373	0.378	Statistically significant relationship with higher education (p=<0.00) Mixed literature findings [16,17,26,65] Conceptual model: Type of role	Exclude
**Training Characteristics**					
COVID-19 training covered 5 or more key topics - binary	0.030**	0.037**	0.001***	Some literature on COVID-19 training generally [17,24,25,65,66] Conceptual model: Training curricula/ intensity	Include
COVID-19 training topics: i) IPC procedures in facility ii) Proper use of Personal Protective Equipment (PPE) iii) Transmission routes iv) Case screening in facility v) Case management (home-based care, referrals, treatment) vi) Risk communication and community engagement (RCCE) vii) COVID-19 Vaccine dissemination procedures viii) Procedures for continuing essential health/ nutrition services ix) Identifying vulnerable individuals in facility or community x) Case testing/lab procedures xi) Disinfection and waste management in facility xii) Community surveillance practices xiii) Case reporting to Ministry of Health xiv) PPE supply chain management	i) 0.035** ii) 0.002***	i) 0.053* iii) 0.026** iv) 0.052* vii) 0.003*** x) 0.079*	ii) 0.061* iii) <0.001*** iv) 0.005*** vi) 0.024** vii) <0.001*** ix) 0.007*** x) 0.006*** xi) 0.041** xii) 0.007*** xiii) 0.007*** xiv) 0.023	Some literature by topic Training topics included were also statistically significant when applied to other outbreaks, and had sufficient empirical diversity.	Include: ii) Proper use of PPE; iii) Transmission routes; iv) Case screening in facility
Post-training very high knowledge rating - binary	**–**	0.000***	<0.001***	Conceptual model: Proxy for adoption, implementation outcome	Include
Health workers who report gaps in pandemic capacity – binary	0.697	0.089	0.051*	Tested against gender for conceptual model-equity considerations, no relationship (p= > 0.05); inverse observation for confidence	Exclude
High satisfaction rating of COVID-19 training -binary	0.446	0.005***	0.014**	Limited literature [19] Conceptual model: Satisfaction, service outcome	Include
**Practice Setting**					
Refresher training or supervision received after initial COVID-19 training – binary	0.041**	0.329	0.819	Limited literature [18]; inverse observation for knowledge Conceptual model: Penetration, implementation outcome	Exclude
Access to PPE - binary	1.000	0.718	0.958	Supporting literature [15,16,67] Conceptual model: Facility resources/supplies	Exclude
Team structure/ staffing considered supportive or efficient (to be prepared for pandemics) - binary	0.096*	0.027**	0.089*	Limited literature [24,68] Conceptual model: Team structure/culture	Exclude
Primary healthcare services continuity post project funding - binary	0.450	0.340	0.035**	Conceptual model: Service continuity	Include
**Ecological System**					
Conflict or insecurity *not* causing restricted access or shutdowns (inverse variable of facility barriers to preparedness)	0.125	0.095*	0.043**	Conceptual model: Facility institutionalization of preparedness protocols	Include
Role change/ promotion since pandemic - binary	0.018**	0.025**	0.248	Conceptual model: Health system job opportunities	Exclude
Services *not* heavily affected by concurrent natural disaster - binary	0.853	0.833	0.043**	Conceptual model: Facing other emergencies, and facility preparedness protocols	Include
Health worker applied five or more COVID-19 training topics to other outbreaks	0.066*	0.060*	0.242	Limited literature [65]	Exclude
COVID-19 training capacities applied to other outbreaks: i) IPC procedures in facility ii) Proper use of PPE iii) Transmission routes iv) Case screening in facility v) Case management (home-based care, referrals, treatment) vi) Risk communication and community engagement (RCCE) vii) COVID-19 Vaccine dissemination procedures viii) Procedures for continuing essential health/ nutrition services ix) Identifying vulnerable individuals in facility or community x) Case testing/lab procedures xi) Disinfection and waste management in facility xii) Community surveillance practices xiii) Case reporting to Ministry of Health xiv) PPE supply chain management	xi) .077*	ii) 0.026** iii) 0.019** iv) 0.041** v) 0.093* vi) 0.040** vii) 0.024** viii) 0.066* ix) 0.035**	ii) 0.075* iii) <0.001*** iv) 0.027** v) 0.072* vii) 0.069*	Limited literature – see above Conceptual model: Facing other outbreaks	Included topics were also statistically significant for COVID-19 training row above

P-value: *p < 0.1, *p* < 0.05 **, *p* < 0.01 ***

^a^Variables not shown in table: Predictors tested but not significant at p < 0.10 for any outcomes and theoretical justification not strong enough to consider for Confidence model: Clinical versus non-clinical role binary; Medical Doctor binary; Gender binary; Post-training assessment binary; Health workers who report being supported by facility/organization to be prepared for future pandemics binary (Sustainability-implementation outcome); Training modality (in-person, remote, hybrid); Training duration; Number of months since the training; Experienced other outbreaks since COVID-19; Health workers that applied all COVID-19 training topics to other outbreaks; Type of health facility (PHC center, mobile clinic, both); Health workers perception that vulnerable community members’ needs were mostly met binary; COVID-19 training topics: IPC procedures in community facility or school (handwashing, screening, isolation) / Mental health and psychosocial support techniques for frontline workers

^b^The “include” variables indicate those that were then tested for the CNA Confidence model, discussed in the next section.

The analyses revealed several significant predictors of high sustained knowledge as perceived by the health workers. In terms of health worker characteristics, more years of experience-binary (*p* = 0.042) was associated with high current knowledge. Related to the training intervention, COVID-19 training covering five or more key topics (*p* = 0.030) and applying these topics to other outbreaks supported a sustained high level of knowledge. Among specific training topics, proper use of PPE (*p* = 0.002) and IPC procedures in the facility (*p* = 0.035) emerged. The practice setting was associated with sustained knowledge when the team structure was considered supportive (*p* = 0.096) and when a role change occurred since the pandemic (*p* = 0.018)—a proxy for ongoing work opportunities within the local health system.

For high sustained skills to face emerging disease risks, as reported by the health workers, many significant predictors matched that of knowledge, which included: more years of experience, supportive health team structure, role change since the pandemic, the COVID-19 training covering five or more topics, and applying those topics to other outbreaks. The sustainment of skills years after training was strongly associated with post-training high knowledge ratings (*p* = 0.000) and high satisfaction with the COVID-19 training (*p* = 0.005). Additionally, specific training topics such as transmission routes (*p* = 0.026), case screening in the facility (*p* = 0.052), COVID-19 vaccination procedures (*p* = 0.003), among others, were associated with the sustained skills outcome.

The sustained high confidence outcome shared many of the same predictors as above for knowledge and skills, including: post-training high knowledge ratings, supportive health team structure, and COVID-19 training covering five or more topics. As with the skills outcome, sustained confidence was also associated with high satisfaction with COVID-19 training (*p* = 0.014). System and practice setting factors such as services not heavily affected by concurrent natural disasters (*p* = 0.043) and conflict or insecurity (*p* = 0.043) were linked to confidence; this is also shown through the predictor of continuity of primary healthcare services post-project funding (*p* = 0.035).

The open-ended survey question on service continuity post-funding revealed that mobile health teams, availability of supplies and personnel, and some primary and secondary services in the health centers were negatively affected or closed when projects ended (see quotes below, Table 4). Among specific COVID-19 training topics, proper use of PPE (*p* = 0.061), transmission routes (*p* < 0.001), case screening in facilities (*p* = 0.005) were associated with current confidence and also reportedly applied to other outbreaks. Other significant training topics for sustained confidence included risk communication and community engagement (RCCE) and social listening (*p* = 0.024), COVID-19 vaccine dissemination procedures (including integration into routine vaccination systems) (*p* = 0.000), among others, from community surveillance practices (*p* = 0.007) to case reporting to the Ministry of Health (*p* = 0.007).

**Table 4:** Relevant quotes from health worker open-ended question responses.

Comment Topic:	Honduras	Syria (GOS/NS)	South Sudan
**Post-funding service continuation**	*“Yes, house to house health and nutrition services, including supplements for malnourished children, these activities ended.”*	*“No, the project ending did not affect services. On the contrary, it gave a very important warning of the need to take measures and develop future plans in order to quickly respond in the event that it appears again.” ~ NS*	*“Facilities closed and this affected the continuation of services to the communities, because no other organization replaced these services.”*
**COVID-19 training satisfaction**	*“Very Satisfied: Because I was kept up to date with a new disease, I was facing the pandemic with knowledge.”*	*“Very Satisfied: We learned about the mechanisms of disease transmission and how to prevent it, we learned communication skills, we learned the types of vaccines and how to administer.” ~ NS*	*“Satisfied: I am satisfied that in the near future it will help me to overcome any kind of pandemic.”*
**COVID-19 training characteristics**	*“We learned a lot; the many topics were quite complete and very informative.”*	*“In my opinion, an interactive training is better.” ~ GOS* *“There should be more topics.” ~ GOS*	*“I learned all the topics and was able to train others.”*
**Perception of team and facility support**	*“Yes, more than a work team, we are a family.”* *“No, because there is no basic equipment.”*	*“Yes, in terms of providing training and an encouraging work environment.” ~ GOS* *“Yes… the spirit of teamwork within the organization gives great support to be among the first ranks to confront any pandemic.” ~ NS*	*“Yes, I feel supported doing my work with [INGO]; they build my capacity through on-the-job training and have paid my incentive/salary.”*
**COVID-19 practices institutionalized by facility**	*“Patients are still asked to wear masks and always try to have hand gel, water and soap in the health center.”*	*“Supervisors follow up and provide us with new updates on a weekly basis.” ~ GOS* *“Continuing to sterilize the center.” ~ NS*	*“Hand washing, use of face masks, and hand sanitizer as part of integrated action in health facility.”* *“Integrated COVID-19 vaccination into the routine vaccination.”*
**COVID-19 training applied to other outbreaks**	*“Triage continues to be used as a filter…for dengue and malaria now.”*	*“Integrating training into daily work…on the type of disease that can spread, such as leishmaniasis and hepatitis, before it occurs.” ~ NS*	*“We were trained for the cholera response as well…how to predict an outbreak.”*

### Health worker quotes on key predictors

The Table 4-Table 5 below provide comments from the health workers where open-ended responses were available related to the key predictors, with selected quotes to further elucidate the survey results.

**Table 5:** Health worker quotes on confidence.

**What influences your feeling confident to apply what you learned from COVID-19 trainings?**
*“Apply knowledge in practice from the beginning, train constantly.” ~ Honduras* *“Supportive leadership, experience, and because I love what I do.” ~ Honduras* *“Fear of incorrect application of some instructions due to lack of training…..Training topics are few.” ~ GOS* *“My self-confidence has become more than before, especially when I apply what I have learned.” ~ NS* *“The source of the information we received was a source highly trusted by all people, which gives us high confidence in ourselves while providing information, and then increasing our communication skills and knowledge of dealing with all segments of society.” ~ NS* *“Because through trainings I learned that COVID-19 was real, and I can apply preventive measures to protect my community from any outbreak of disease.” ~ South Sudan*

Open-ended questions allowed health workers to provide more description of the factors that influence their confidence to face other infectious disease threats (Table 5). Most health worker responses note themes around their confidence stemming from experience and practice, as well as having adequate knowledge from trusted sources. Some health workers note the importance of becoming good health communicators, but also that the lack of response or uptake of preventive behaviors from their patients or community dampens their confidence.

### Coincidence analysis for confidence model

The predictor association results were then used to build the Confidence model for the CNA. The final column in Table 3 above indicates “include” for the predictors with significant associations with the sustained high confidence outcome and/or substantial theoretical justification based on literature or relevance to the conceptual framework. These variables were then tested for redundancy and association with the Confidence outcome using chi-square tests, pairwise correlation analyses, and logistic regression (*p* = 0.05) in order to reduce the number of predictors for the CNA model to six. The Confidence model used for the CNA included the following six predictors covering key Dynamic Sustainability Framework domains:

1) Individual Characteristic – Higher education (University or higher completed);
2) Intervention Characteristic - COVID-19 training covered five or more key topics,
3) Intervention Characteristic - Training topic on case screening in the facility;
4) Implementation outcome - Post-training very high knowledge (proxy for adoption);
5) Practice/System setting - Service continuity post-funding;
6) Practice/System setting - Services not highly affected by natural disasters during the pandemic.

The CNA for the presence of current very high confidence (*Outcome*) identified multiple causal pathways leading to the confidence outcome, reflecting equifinality (Table 6). Two primary configurations or solutions emerged as sufficient for sustained confidence. The first configuration indicated that post-training very high perceived knowledge (*Predictor 4*) was sufficient on its own to lead to sustained confidence to face future pandemics (consistency: 66.0%; coverage: 62.0%). This means two-thirds of the cases with very high perceived knowledge (66%) showed sustained confidence (i.e., how reliably this condition leads to the outcome), while this configuration accounts for 62% of all cases where sustained confidence occurred (i.e., how much of the outcome it explains).

**Table 6:** CNA sufficient conditions solutions.

Outcome	Condition/Predictor		Consistency	Coverage	Complexity	INUS
1 Very High Confidence	Post-training Very High Knowledge		0.660	0.62	1	TRUE
2 Very High Confidence	College Education *	Case Screening Training Topic	0.604	0.64	4	TRUE
		Post-funding Service Continuity				

Note: Table developed to replicate CNA final solutions using full variable descriptions instead of variable names.

The second solution set demonstrated conjunctural causation, meaning the outcome arises from a combination of predictors together (Table 6). This solution indicated that very high confidence was associated with either the training topic of case screening at the facility was received *(Predictor 3)* in combination with the health worker having a higher education level (*Predictor 1*), or if PHC services continued post-funding *(Predictor 5)* for health workers with higher education levels *(Predictor 1)*. This second pathway had a consistency of 60.4% and a coverage of 64.0%. Predictor 6 of the model did not appear in the CNA solutions. See Limitations for further discussion of the consistency and coverage rates below the estimated threshold (0.8).

No single condition was identified as necessary for the outcome. This means that no single factor was always present when the outcome occurred, as the outcome can arise from different combinations of conditions rather than one essential cause. All three solutions were considered INUS, that is, “Insufficient but Necessary part of a condition which is itself Unnecessary but Sufficient” [61].

## Discussion

### Overview

This study sought to identify the necessary and sufficient factors, including COVID-19 training implementation strategies and contextual factors, associated with sustained health emergency capacities over time amongst surveyed PHC workers from the humanitarian contexts of Honduras, Syria (GOS and NS areas), and South Sudan. The study first assessed predictors associated with the capacity outcomes of current “very high” self-rated knowledge, skills, and confidence to face future outbreaks or pandemics. These included predictors related to health worker individual and intervention characteristics, implementation and service outcomes, practice setting, and other ecological system factors per domains of the Dynamic Sustainability Framework.

The analyses proceeded to develop a model for the sustained very high confidence outcome to test causal pathways using CNA. With no single ‘necessary’ factor, the CNA results highlight the presence of multiple sufficient pathways leading to sustained confidence to face pandemics. The causal conditions contributing to the presence of sustained confidence included post-training knowledge and combinations of key training topics, service continuity, and health worker education level. The findings highlight the critical role of tailored and comprehensive training content, considering training participant backgrounds, and contextual and practice setting factors in sustaining health worker preparedness capacity.

This discussion outline follows the key domains of individual and intervention characteristics and the practice setting and broader context. It continues with a discussion of the application of COVID-19 capabilities by humanitarian health workers to other outbreaks facing the world today or in the future and with a discussion of policy implications. The section ends with further description of study limitations.

### Individual and intervention characteristics

The CNA revealed multiple sufficient pathways to sustained confidence. The individual characteristic of health worker education level is a key condition in the CNA solutions. While higher health worker education pre-training was not statistically significantly associated with the outcomes, it was included in the model as substantiated by the literature and because it was significantly correlated with type of clinical role. Existing literature from COVID-19 consistently links higher health worker education levels to improved knowledge, skills/practice, or confidence/readiness in disease outbreak response and IPC measures [15,16,19,63–65,69]. This is also supported by a conceptual framework of health professional confidence, in which existing expertise and authority is a key component [22].

Intervention factors statistically associated with high sustained pandemic capacities (health worker knowledge, skills, and confidence) included comprehensive COVID-19 training covering five or more key topics, applying training topics to other outbreaks, and specific technical content such as proper PPE use, transmission routes, and case screening procedures in the facility. Health worker perceptions of their post-training knowledge for emerging disease risks (proxy for adoption) and training satisfaction were also associated with perceptions of sustained skills and confidence. The COVID-19 response prompted a swift increase in capacity building of humanitarian frontline health workers, though the ad-hoc trainings varied greatly in format and curriculum [70]. Rapid and comprehensive training of all health workers has been identified as essential to health system resilience and was a defining characteristic of “high-performing countries” during the pandemic [71]. As quoted in Table 4-Table 5 above, health worker respondents from this study described the importance of the timely and complete curriculum, and a health worker from Honduras noted the “many topics were quite complete.”

The importance of specific training topics, such as case screening in the facility, is supported by evidence associating COVID-19 case management knowledge and various facility IPC measures with increased confidence and effective workplace practices [15–17,19,24,72]. Comprehensive training that includes key COVID-19 topics has been consistently associated with higher KAP (knowledge, attitude, practice) and self-efficacy scores in various humanitarian settings [17,24,25,65,66]. These findings reinforce the necessity of institutionalizing health emergency training and protocols, while also considering how health worker role or educational background may influence the uptake and retention of capacities [22]. It further confirms the need for GHS competencies to be developed for all levels of the health workforce, including in fragile and low-resource settings [70,73,74].

Despite the established literature highlighting good practices in health worker in-service training design and delivery [75], training modality and duration did not emerge as significant associations in this study, nor were they key factors in the Confidence model analyses. Prior research underscores the importance of interactive, iterative learning methods, particularly when implemented near clinical settings [75]. The divergence from this literature may reflect the high level of variation across the trainings received by respondents [70].

### Practice setting and ecological context

The main practice setting factor that significantly contributed to sustained confidence was supportive team and staffing structures. The importance of supportive team structures for health system resilience and preparedness at the frontlines is well documented [6,71,76]. Health worker competence and confidence are linked to the “relational dynamics between individuals working together” [22]. Health workers’ own words from this study capture this dynamic, describing their team as a “family” or “the spirit of teamwork.”

This study also analyzed health worker access to follow-up training or supervision (penetration) and perceived ongoing support from their organization to be prepared for future pandemics (sustainability) as implementation outcomes for institutionalizing preparedness capacities. While these measures were not included in the CNA confidence model due to a ack of statistically significant associations, access to refresher training or supervision was associated with sustained knowledge. The combination of IPC training with supportive supervision, workplace culture reinforcement, and effective information dissemination has been shown to enhance learning outcomes [77], and is considered good practice for in-service training [75]. Studies in Uganda have similarly demonstrated the value of modular, participatory training approaches supported by mentoring or supervision to improve knowledge retention and application [78,79]. In fact, in open-ended questions for this survey some health workers responded to the question of feeling supported by their facility by naming their access to trainings, and others described the institutionalization of IPC practices in their facilities. The absence of further significant associations for these institutionalization measures with the capacity outcomes may reflect issues around the proxy measures that should be explored in future study [32]. It may also show an interplay with more pressing contextual factors, such as safety and wellbeing for health workers and their families [76], other health facility characteristics, or a potential misalignment between post-training monitoring and support and the specific needs of health workers in humanitarian settings [18,64,80]. As these conditions were not explored in this study, they indeed merit further research.

Contextual factors that significantly contributed to sustained confidence were health service continuity after the end of project funding and minimal disruptions from natural disasters or conflict. The health workers of this study described the home visits, mobile clinics, and facilities that were reaching isolated communities with the surge of COVID-19 grants and subsequently shut down post-funding. Health workers thus lose confidence when community access to services is beyond their control, because “no other organization replaced these services,” as noted by a respondent from South Sudan. The cases for this study represent three difference complex-emergency contexts. Health workers in Honduras were managing COVID-19 response while simultaneously responding to Hurricanes Eta and Iota (2020), which displaced populations, damaged health facilities, and disrupted supply chains. In Syria, the 2023 earthquake further strained a health workforce already operating under protracted conflict and fragmented governance. In South Sudan, recurrent flooding and chronic insecurity interrupted facility operations and staff deployment. These compounding emergencies illustrate how non-pandemic disasters can undermine the enabling conditions that this study identifies as essential to sustained confidence. Literature on health system resilience during the pandemic emphasizes the importance of financial and social supports for health workers and structures to ensure primary care is equipped to respond and to continue services [6,71].

Humanitarian health systems face chronic challenges around severely limited access to PPE and other supplies, workforce development systems, and surveillance and laboratory systems [10–12,81]. Literature from COVID-19 in humanitarian settings documents the importance of combining training with health worker access to the PPE or other equipment necessary to support their capacities to deliver services [18,64,82]. An assessment of COVID-19 training curricula in humanitarian settings found an absence of training related to continuity plans for essential services [70]. Frontline health workers across Uganda surveyed early in the pandemic reported severe shortages of PPE and other facility infrastructure to provide isolation or oxygen support [83]. Shortages of PPE, along with barriers like limited facility space and understaffing, have been reported to undermine confidence in applying training knowledge [64,84]. For this study, the training topic on proper use of PPE was important, however, access to PPE was reportedly not a significant predictor of the outcomes as recalled by health workers. This divergence from the literature could be explored through future study.

While the consistency and coverage thresholds for the CNA solutions were lower than desired and this should be considered in the interpretation of the findings, such thresholds are just one tool for model development [85]. This study provides associations and predictors that can be refined and expanded for future models on humanitarian health worker preparedness, and it further demonstrates the use of CNA [55], including for implementation research studies in complex contexts that lack causal research [86,87]. Individual, intervention, and contextual factors not captured in the survey but that arose from some open-ended responses and that may be particularly relevant include: health worker mental health and burnout, local community trust in health services, and facility-level leadership dynamics, all of which likely shape the enabling environment for sustained confidence but were beyond the scope of this study’s survey instrument.

### COVID-19 capabilities applied to other outbreaks

The application of various COVID-19 training topics to other concurrent outbreaks were associated with higher health worker knowledge, skills, or confidence to face these threats. Concurrent health threats faced by the sampled health workers included dengue in Honduras, and numerous infectious disease outbreaks such as cholera and measles in Syria and South Sudan. The findings resonate with evidence that previous outbreak experience, and the application of COVID-19 training to other outbreaks, were associated with higher outbreak response awareness scores [65].

Contexts with low-resource and weak health systems are the most probable “spark” settings for future outbreaks or pandemics [88]. Future health emergency preparedness must account for the likelihood of emerging pathogens distinct from SARS-CoV-2, with zoonotic spillover events predicted to rise exponentially in coming decades due to climate change and habitat encroachment [4]. This includes, for example, the continued spread of highly pathogenic avian influenza A (H5N1) viruses and the potential for novel strains with human-to-human transmissibility, which have shown “explosive” transmission among birds across continents in recent years [89–91]. Beyond viruses, the “silent pandemic” of antimicrobial resistance poses increasing global risks, exacerbated by climate emergencies and humanitarian crises [92], which includes drug-resistant Tuberculosis [93].

The training topics most associated with health worker knowledge, skills, or confidence in this analysis, such as proper PPE use, RCCE, case screening, and facility IPC measures, are pathogen-agnostic and directly relevant to mitigating risks posed by future pathogens [94]. COVID-19 response capacities may be applied to all future pathogens where health facility IPC and triage measures, community trust and surveillance, and responder capacities are critical to reducing transmission and mortality.

### Policy implications

This study informs the GHS and resilient health systems agendas for frontline health workers in humanitarian settings. Specific investment in ongoing capacity building for frontline health workers in humanitarian contexts will be essential to ensure they can continue to implement critical knowledge and skills in the face of future global health emergencies. Emerging hybrid financing models that blend public and private capital could provide opportunities to stabilize funding and build more resilient health systems in fragile contexts, provided they complement rather than replace official development assistance [95]. The study should also inform the policy discussions to better integrate strategies for pandemic preparedness and response and Universal Health Care, both acknowledging the importance of strong PHC systems [96,97].

### Limitations

This study has several limitations that warrant consideration. Selection bias due to non-response and staff turnover in humanitarian settings may have led to over-representation of retained health workers with higher capacity. As described above, verification with local partners that individual characteristics of the sample did not differ from the full sample frame aimed to mitigate this risk. Nevertheless, health workers in more accessible facilities were more likely to be included due to security or logistical constraints. This form of selection bias is consistent with a broader challenge documented in health workforce research in humanitarian settings, where systematic exclusion of hard-to-reach facilities and populations may produce estimates that reflect more stable or functional sub-contexts rather than the full operational range [98]. This limitation should be considered when interpreting the capacity estimates.

Self-assessment measures are subject to biases such as recall bias, social desirability bias, and the Dunning-Kruger effect, where less-skilled individuals may overestimate their capabilities. Privacy assurances and contextual reminders during surveys sought to improve recall accuracy, while qualitative data with staff and health partners were used to validate self-reports [99,100]. Notably, confidence ratings measured years after training allowed for benefits of retrospective recall, which argues this approach may offer a more accurate reflection of retained expertise compared to immediate post-training assessments [99,101,102].

Finally, the cross-sectional design limits internal and external validity. While still constrained, the CCM approach employed enhances internal validity by integrating qualitative data and detailed case knowledge [103]. For external validity, findings may not be generalized across all humanitarian settings due to the cross-sectional and non-probability design, which provides a descriptive estimate of capacity within the sampled population [104]. In addition, the lower consistency and coverage rates of the CNA solutions and absence of necessary conditions show that the model was missing key predictors to more fully explain sustained confidence and that data quality (noise) may have affected results [57]. Unobserved confounding factors may have also influenced the coverage results, as reported for the CNA method [56]. The purposive selection and extensive familiarity with the diverse country cases supports the assumption that confounders are distributed equally across the cases [55]. The prominence of the health worker educational background predictor in the solutions indicates future study should sample based on education and/or clinical role.

## Conclusions

This study uses statistical correlations and coincidence analysis to identify critical factors for sustaining frontline health worker future outbreak or pandemic preparedness in the humanitarian settings of Honduras, Syria, and South Sudan. Key findings emphasize the importance of comprehensive training including key topics such as proper use of PPE, transmission routes, case screening, and IPC measures in the health facility. These topics are transferable to responses to subsequent outbreaks and are relevant to threats such as mpox and H5N1 viruses with pandemic potential. Key predictors of sustained health worker confidence to face emerging disease threats also included health worker education level and the importance of supportive health teams in the practice setting. These results underscore the need for targeted investments in ongoing outbreak and pandemic preparedness and response training and health system resilience, particularly in humanitarian contexts where health service disruptions from unpredictable funding cycles, conflict, or natural disasters undermine workforce readiness.

Policies and initiatives for Global Health Security should prioritize capacity-building efforts that align comprehensive training content with local needs and across health worker roles, strengthen supportive supervision and outbreak knowledge dissemination, and ensure primary healthcare service continuity to mitigate future pandemic risks. Future research should further explore the integration of comprehensive health emergency training within humanitarian health systems and adequate measures to fully explore health worker confidence and competence to face future pandemics.

## Supporting information

S1 Fig CNA Examination of sufficiency predictor patterns for confidence outcome. (TIF)

S1 Data Health worker survey data. The underlying dataset anonymized.
(XLSX)

S1 Checklist Inclusivity in global research. Questionnaire.
(DOCX)
