# Supplementary material for: Coincidence analysis: Identifying factors contributing to frontline health workers’ confidence to face future pandemics across Honduras, Syria, and South Sudan
Source: PLOS Glob Public Health. 2026 Jul 31;6(7):e0006925. doi: 10.1371/journal.pgph.0006925 (PMC13426930; doi:10.1371/journal.pgph.0006925)
Supplement: Attachment — Submitted filename: Reviewer Response.docx [file pgph.0006925.s004.docx]

| **Comment** | **Section** | **Response** |
| --- | --- | --- |
| **Journal Requirements:** | | |
| Please include a complete copy of PLOS’ questionnaire on inclusivity in global research in your revised manuscript. | Methods (circa p.8) and Supplementary Information | Uploaded as Supplementary Information S3_Checklist and referenced in Methods main body as the policy suggests. |
| COI Statement |  | Attached as separate document |
| Data | Supplementary Information | Uploaded as Supporting Information S2_Data |
| Figure Resubmission/NAAS Tool | Fig 1, S1_Fig | The ‘fixed’ versions have been resubmitted. See the screenshot at end of this document confirming the Naas tool has fixed the two figures. |
| **Review Comments to the Author:** | | |
| Introduction need to address specific knowledge gap; it is not properly addressed. | Introduction | Thank you, the Introduction section has been heavily revised for clarity, especially the first paragraph. The first sentence notes gaps, and the final sentence of the first paragraph states the ‘aim’ of the research. |
| In table-1, % with >1 dose of COVID-19 vaccine is very less (27%, 18%), this needs further explanation | Table 1 | Thank you, updated Table 1 for clarity and added a descriptive paragraph above, including a cited sentence on the low vaccination rate in Syria. |
| In Methodology sampling design (appears to be purposive), participants inclusion and exclusion criterias are not defined.  No details regarding training, standardization and supervision of data are provided. Details needed.  Details regarding any measure were taken to decrease selection bias (if taken) should be provided. | Methodology | Added sub-headings to better organize Methods section. Edited and added clarity to the sampling, including purposive design for the country and site selection. And for the participant selection, clarified the inclusion/exclusion criterion.  More detail on enumerator training, standardization and supervision of data quality added just before Analysis Procedures in a new “Data Collection and Management” section.  Measures to mitigate selection bias further explained in participant selection section and in Limitations. |
| Kindly discuss confounders adequately. It needs more elaboration. | Results/Discussion/  Limitations | Added sentence to CNA results section. Added a few sentences to Discussion, and then expanded Limitations. CNA has particular indications for considering confounders that has been added to the Limitations section, where it seemed most relevant to discuss – and further citations added. |
| Discussion is mostly repetition of the results. Kindly discuss it with more relevant studies. | Discussion | Thank you, and agreed. The Discussion section has been reorganized completely, and it now focuses more on discussion of results in relation to literature. |
| Some paragraphs are unnecessary lengthy. Kindly reframe.  Acronyms should be elaborated at first use. | Throughout manuscript | Absolutely correct. Edited entire document for clarity. Revised sentences and paragraphs that were too lengthy.  Acronyms checked. |
| Selection bias and generalisability need more elaboration. | Limitations | Added additional sentences and citations for each of those topics in the Limitations section. |

Figure Resubmissions: While revising your submission, we strongly recommend that you use PLOS’s NAAS tool (<https://ngplosjournals.pagemajik.ai/artanalysis>) to test your figure files. NAAS can convert your figure files to the TIFF file type and meet basic requirements (such as print size, resolution), or provide you with a report on issues that do not meet our requirements and that NAAS cannot fix.

After uploading your figures to PLOS’s NAAS tool - <https://ngplosjournals.pagemajik.ai/artanalysis>, NAAS will process the files provided and display the results in the "Uploaded Files" section of the page as the processing is complete. If the uploaded figures meet our requirements (or NAAS is able to fix the files to meet our requirements), the figure will be marked as "fixed" above. If NAAS is unable to fix the files, a red "failed" label will appear above. When NAAS has confirmed that the figure files meet our requirements, please download the file via the download option, and include these NAAS processed figure files when submitting your revised manuscript.
