# Supplementary material for: Coincidence analysis: Identifying factors contributing to frontline health workers’ confidence to face future pandemics across Honduras, Syria, and South Sudan
Source: PLOS Glob Public Health. 2026 Jul 31;6(7):e0006925. doi: 10.1371/journal.pgph.0006925 (PMC13426930; doi:10.1371/journal.pgph.0006925)
Supplement: Attachment — Submitted filename: PLOS REVIEWERS.docx [file pgph.0006925.s005.docx]

**PLOS REVIEWERS’ COMMENTS:**

**Title: Coincidence analysis: Identifying factors contributing to frontline health workers' confidence to face future pandemics across Honduras, Syria, and South Sudan** **Manuscript Number:** **PGPH-D-25-03335R1**

**General Overview**

This is a timely and important study that addresses a critical gap in our understanding of how to sustain pandemic preparedness among frontline health workers in humanitarian and fragile settings.

By looking back two to three years after COVID-19 training, the authors provide valuable insights into what actually "sticks" over time in challenging environments like Honduras, Syria, and South Sudan. The use of Coincidence Analysis (CNA) is an innovative choice that successfully highlights the complex, overlapping pathways that lead to health worker confidence.

Using configurational comparative methods (CNA) allows the study to move beyond simple linear associations to show how various factors like education and specific training topics work together to produce results.

The manuscript can be considered for publication after the following minor revisions.

**Areas for revision:**

In the abstract, please structure section into background; methods, results etc …and summarize what you did under each subheading.

Line 32-34,in the abstract; there are dashes suggestive of using AI in writing kindly rephrase and re align accordingly

While the paper does an excellent job of describing the three different regions, some readers might benefit from a clearer link between the specific "emergency types" (e.g., the hurricanes in Honduras or the earthquake in Syria) and how those specific events directly impacted the health workers' ability to apply their training.

Kindly consider including some studies that looked at frontliners readiness during the covid 19 pandemic for example:

[https://doi.org/10.24248/eahrj.v5i2.665 /](https://doi.org/10.24248/eahrj.v5i2.665%20/) <https://pmc.ncbi.nlm.nih.gov/articles/PMC8751497/> <https://doi.org/10.24248/eahrj.v5i2.665>

The authors honestly note that the CNA solutions had lower consistency and coverage thresholds than is often ideal. To strengthen the paper, it would be helpful to expand the discussion on what other "unobserved" factors might have been at play for example mental health, local community trust, or specific facility level leadership styles that weren't fully captured in the survey.

The qualitative quotes included in the results (such as the worker from South Sudan realizing "COVID-19 was real") are incredibly impactful. Including a few more of these voices in the Discussion section could further illustrate the "relational dynamics" the authors mention as being key to teamwork.

Authors correctly identifies the risks of recall bias and the Dunning-Kruger effect in self-assessment. It would be beneficial to explicitly state how the "privacy assurances" and "contextual reminders" mentioned were implemented during the survey to reassure readers about the steps taken to mitigate these biases.
