# Supplementary material for: Coincidence analysis: Identifying factors contributing to frontline health workers’ confidence to face future pandemics across Honduras, Syria, and South Sudan
Source: PLOS Glob Public Health. 2026 Jul 31;6(7):e0006925. doi: 10.1371/journal.pgph.0006925 (PMC13426930; doi:10.1371/journal.pgph.0006925)
Supplement: Attachment — Submitted filename: Response to Reviewers_12Jun.docx [file pgph.0006925.s006.docx]

**Response to Reviewers**

Dear *PLOS Global Public Health* editors and reviewers,

Thank you for your thoughtful comments regarding the paper below:

PGPH-D-25-03335R1

“Coincidence analysis: Identifying factors contributing to frontline health workers' confidence to face future pandemics across Honduras, Syria, and South Sudan”

We include this document to respond to the final reviewer comments (7 total). We believe all comments have been addressed. The track-changed manuscript and clean manuscript are attached separately. We wanted to extend our gratitude for your thoughtful inputs and support in this publication process.

We submit this revision **in advance** of the deadline: 29 June 2026 11:59PM

Best regards,
Dr. Christopher Kemp (Corresponding author)

| **Comment** | **Section** | **Response** |
| --- | --- | --- |
| **Journal Requirements:** | | |
| No further comments |  | N/A |
| **Review Comments to the Author:** | | |
| 1. In the abstract, please structure section into background; methods, results etc …and summarize what you did under each subheading. | Abstract | The PLOS formatting guidelines do not allow for abstract sub-headings and this is not standard practice in the published research articles for this journal. Therefore, we did not add sub-headings, but we did review and edit the abstract to ensure it is ordered appropriately. |
| 2. Line 32-34,in the abstract; there are dashes suggestive of using AI in writing kindly rephrase and re align accordingly | Abstract | We have rephrased the sentence at lines 32–34 of the abstract to remove the em-dash construction flagged by the reviewer, replacing it with a colon and then listing the specific topics. This also corrects a subject-verb agreement error in the original phrasing. |
| 3. While the paper does an excellent job of describing the three different regions, some readers might benefit from a clearer link between the specific "emergency types" (e.g., the hurricanes in Honduras or the earthquake in Syria) and how those specific events directly impacted the health workers' ability to apply their training. | Discussion, "Practice setting and ecological context" section | We have added language to the Discussion section to explicitly link the specific disaster events in each country (the Honduras hurricanes, Syria earthquake, and South Sudan flooding) to the mechanisms by which they affected health workers' ability to apply their training. This connection was previously implicit and we agree it strengthens the paper's contextual grounding. This sub-section has also been reorganized and edited for better flow. |
| 4. Kindly consider including some studies that looked at frontliners readiness during the covid 19 pandemic for example:  [https://doi.org/10.24248/eahrj.v5i2.665 /](https://doi.org/10.24248/eahrj.v5i2.665%20/) <https://pmc.ncbi.nlm.nih.gov/articles/PMC8751497/> <https://doi.org/10.24248/eahrj.v5i2.665> | Discussion, "Practice setting and ecological context" section | Thank you for sharing your interesting research. In the same section as the comment above, we have added a sentence that references your paper: Frontline health workers across Uganda surveyed early in the pandemic reported severe shortages of PPE and other facility infrastructure to provide isolation or oxygen support. |
| 5. The authors honestly note that the CNA solutions had lower consistency and coverage thresholds than is often ideal. To strengthen the paper, it would be helpful to expand the discussion on what other "unobserved" factors might have been at play for example mental health, local community trust, or specific facility level leadership styles that weren't fully captured in the survey. | Discussion, "Practice setting and ecological context" section | At the end of this section, we explicitly name examples of unobserved factors that may have influenced results, including health worker mental health, community trust, and facility-level leadership. These were not captured in the survey instrument and represent important areas for future research, as the reviewer rightly notes. |
| 6. The qualitative quotes included in the results (such as the worker from South Sudan realizing "COVID-19 was real") are incredibly impactful. Including a few more of these voices in the Discussion section could further illustrate the "relational dynamics" the authors mention as being key to teamwork. | Discussion | We have incorporated references back to the qualitative quotes available from the health worker survey. This strengthens the connection between the CNA findings and the lived experience of health workers in these settings, as the reviewer suggests. Thank you! |
| 7. Authors correctly identifies the risks of recall bias and the Dunning-Kruger effect in self-assessment. It would be beneficial to explicitly state how the "privacy assurances" and "contextual reminders" mentioned were implemented during the survey to reassure readers about the steps taken to mitigate these biases. | Methods, “Data collection and management” section | We have added explicit description of the privacy assurance and contextual reminder procedures to the Data Collection section of the Methods. This clarifies the specific steps taken to mitigate recall bias and social desirability bias, which were referenced in the Limitations but not previously described in the Methods. |
